# Role of Hydrogen Sulfide in Oral Disease

**DOI:** 10.1155/2022/1886277

**Published:** 2022-01-25

**Authors:** Dong-Dong Wu, Ebenezeri Erasto Ngowi, Yuan-Kun Zhai, Yi-Zhen Wang, Nazeer Hussain Khan, Ahmad Fadhil Kombo, Saadullah Khattak, Tao Li, Xin-Ying Ji

**Affiliations:** ^1^School of Stomatology, Henan University, Kaifeng, Henan 475004, China; ^2^Henan International Joint Laboratory for Nuclear Protein Regulation, School of Basic Medical Sciences, Henan University, Kaifeng, Henan 475004, China; ^3^Kaifeng Municipal Key Laboratory of Cell Signal Transduction, Henan Provincial Engineering Centre for Tumor Molecular Medicine, Henan University, Kaifeng, Henan 475004, China; ^4^Department of Biological Sciences, Faculty of Science, Dar es Salaam University College of Education, Dar es Salaam 2329, Tanzania; ^5^School of Life Sciences, Henan University, Kaifeng, Henan 475004, China; ^6^Kaifeng Key Laboratory of Infection and Biological Safety, School of Basic Medical Sciences, Henan University, Kaifeng, Henan 475004, China

## Abstract

Oral diseases are among the most common human diseases yet less studied. These diseases affect both the physical, mental, and social health of the patients resulting in poor quality of life. They affect all ages, although severe stages are mostly observed in older individuals. Poor oral hygiene, genetics, and environmental factors contribute enormously to the development and progression of these diseases. Although there are available treatment options for these diseases, the recurrence of the diseases hinders their efficiency. Oral volatile sulfur compounds (VSCs) are highly produced in oral cavity as a result of bacteria activities. Together with bacteria components such as lipopolysaccharides, VSCs participate in the progression of oral diseases by regulating cellular activities and interfering with the immune response. Hydrogen sulfide (H_2_S) is a gaseous neurotransmitter primarily produced endogenously and is involved in the regulation of cellular activities. The gas is also among the VSCs produced by oral bacteria. In numerous diseases, H_2_S have been reported to have dual effects depending on the cell, concentration, and donor used. In oral diseases, high production and subsequent utilization of this gas have been reported. Also, this high production is associated with the progression of oral diseases. In this review, we will discuss the production of H_2_S in oral cavity, its interaction with cellular activities, and most importantly its role in oral diseases.

## 1. Introduction

Oral cavity is an important organ that plays a huge role in social interactions, and therefore, the health of the organ is critical for individual's personality, confidence, and communication. Oral diseases include acute and chronic health problems that target the oral system. The diseases range from discomforts caused by bad smell produced by the cavity to chronic diseases that can occur in the organ. Oral diseases are some of the least studied, and their treatments have not been well established [1, 2]. Hence, there is an enormous need to address the issue and identify possible diagnostic and therapeutic targets for such diseases.

Hydrogen sulfide (H_2_S) is a potent gaseous neuromodulator involved in the regulation of crucial cellular processes such as inflammation, oxidative stress, autophagy, and apoptosis [3, 4]. In many diseases, H_2_S has been reported to be involved in their development and progression [4–6]. The gas is also highly produced in oral cavity and has been numerously associated with the progression of oral diseases. H_2_S is produced both endogenously by oral cells and exogenously through the activities of oral microbes. Understanding the importance of this gas in the progression of oral diseases is of greater importance due to its activities in cell regulation. Therefore, in this review article, we will discuss the molecular mechanisms associated with the production and utilization of H_2_S and correlate the dysregulation of this gaseous neuromodulator with oral diseases.

## 2. H_2_S Production in Oral Cavity

### 2.1. Endogenous H_2_S Production in Oral Cavity

The endogenous production of H_2_S is principally catalyzed by three enzymes, namely, cystathionine gamma-lyase (CSE), cystathionine beta-synthase (CBS), and 3-mercaptopyruvate sulfur transferase (3-MST). The former two are members of the pyridoxal 5′-phosphate- (PLP-) dependent enzymes and are known to be involved in the metabolism of amino acids. CSE and CBS have been reported to be significantly expressed in gingival tissues at both gene and protein levels [7]. At cellular level, the two enzymes have also been identified to be the main producers of H_2_S in human periodontal ligament [8]. 3-MST is also strongly expressed in oral tissues. In oral cavity, H_2_S participates in the regulation of cellular homeostasis. For instance, gingival crevicular fluid (GCF) volume which is an important parameter of oral health has been positively correlated with clinical features such as inflammation [9]. Simultaneously, the GCF volume significantly correlates with both the production of H_2_S by the cervical fluid and the rate of inflammation [10]. The above data confirms the production of H_2_S in oral cavity by different sites and its involvement in oral health.

### 2.2. H_2_S Production by Oral Bacteria

**Figure 1:**
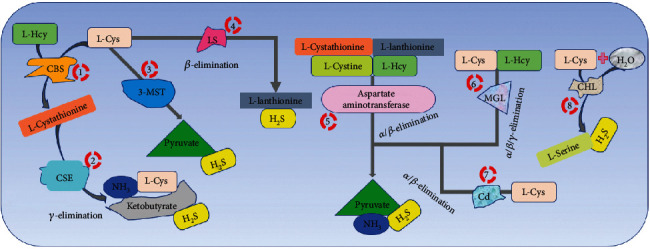
The illustration of the potential mechanisms used by oral bacteria to mediate H_2_S production. From left to right: CBS from bacteria catalyzes the substrates L-Hcy and L-Cys to yield L-cystathionine which is then converted to ketoglutarate, NH_3_, L-Cys, and H_2_S by CSE. Otherwise, 3-MST can also convert L-Cys to pyruvate and H_2_S; meanwhile, LS converts it to L-lanthionine and H_2_S. Other enzymes such as aspartate aminotransferase (substrates: L-cystathionine, L-lanthionine, L-cystine, and L-Hcy), MGL (L-Cys and L-Hcy), CHL (L-Cys+H_2_O), and Cd (L-Cys) catalyze the production of H_2_S, pyruvate, and NH_3_. CBS: cystathionine beta-synthase; L-Hcy: L-homocysteine; L-Cys: L-cysteine; H_2_S: hydrogen sulfide; CSE: cystathionine gamma-lyase; 3-MST: 3-mercaptopyruvate sulfur transferase; LS: lanthionine synthase; MGL: L-methionine gamma-lyase; CHL: cysteine (hydroxyl) lyase; Cd: cysteine desulfurase; NH_3_: ammonia; H_2_O: water.

Apart from endogenous production, bacteria present in the oral cavity synthesize H_2_S through several enzymatic reactions. Some of these enzymes include CSE, CBS, and 3-MST [11–13], cysteine desulfurase (CD) [14–18], lanthionine synthase (LS) [19–23], aspartate aminotransferase [21, 24], l-methionine gamma-lyase (MGL) [21, 25–28], and cysteine hydroxyl lyase (CHL) [21, 29]. Through different mechanisms, these bacteria-contained enzymes catalyze the production of H_2_S from multiple substrates (Figure 1).

## 3. H_2_S-Regulated Cellular Mechanisms in Oral Cavity

### 3.1. Oxidative Stress

**Figure 2:**
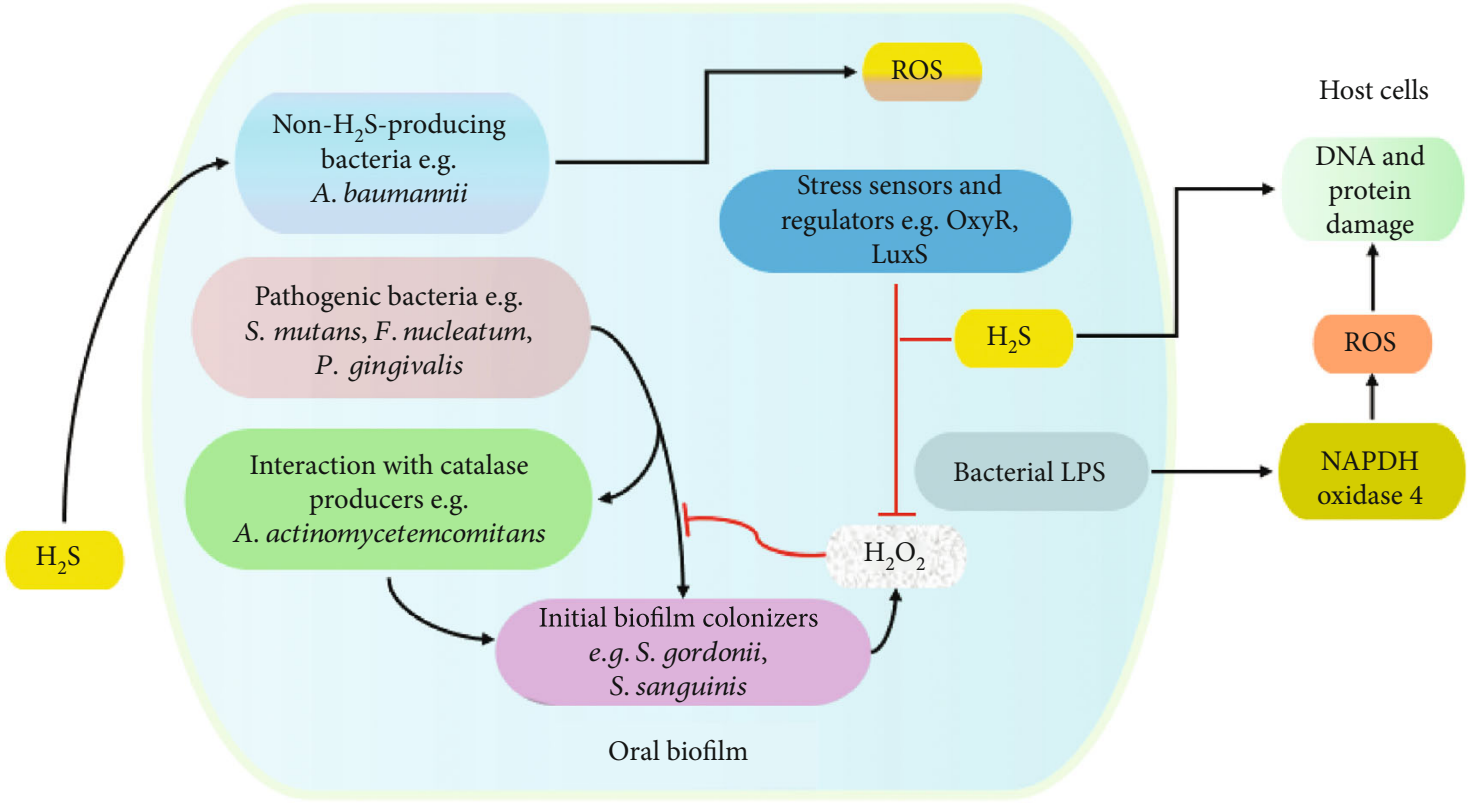
The representation of the possible role of H_2_S in bacterial survival and subsequent cellular regulation of oxidative stress. Initial bacteria colonizers produce H_2_O_2_ to primarily prevent the binding of pathogenic bacteria. The commensal secondary colonizers possess several oxidative stress regulators such as OxyR and LuxS, in addition to H_2_S which together help to prevent the toxicity of H_2_O_2_. However, pathogenic bacteria also contain antioxidant regulators such as H_2_S which can also improve their survival. Besides, the presence of catalase producers on the biofilm can facilitate the binding and survival of pathogenic bacteria. The bacterial-produced H_2_S can increase oxidative stress in host cells via the activation of NADPH4. In the case of non-H_2_S-producing bacteria, exogenous H_2_S elevates the prooxidative status and reduces their survival. H_2_S: hydrogen sulfide; ROS: reactive oxygen species; H_2_O_2_: hydrogen peroxide; LPS: lipopolysaccharides; DNA: deoxyribose nucleic acid; NADPH4: nicotinamide adenine dinucleotide phosphate oxidase 4.

Oxidative stress is one of the commonly dysregulated entities and a potential therapeutic target in chronic oral diseases [30, 31]. Oxidative stress is caused by an imbalance between oxidants and antioxidant levels which result into protein, lipids, DNA, and RNA oxidation and damage. In oral cavity, oxidative stress can be induced by many factors including cigarette smoking [32], metabolic diseases [33], hydrogen peroxide- (H_2_O_2_-) based tooth whitening products [34], food [35], and most importantly from oral bacteria [36–38]. High oxidative stress leads to the promotion of senescence-like features [39] and progression of oral diseases [40, 41]. In human cellular models, the bacterial-produced H_2_O_2_ have been demonstrated to be lethal to both epithelial and macrophage cells [42, 43]. With regard to H_2_S, previous studies indicate that exogenous H_2_S can promote reactive oxygen species (ROS) generation and subsequently DNA damage in both human gingival epithelial cells and keratinocyte stem cells [44, 45]. Meanwhile, in oral bacteria, both pro- and antioxidative properties have been reported in bacteria following H_2_S treatment which suggests that its subsequent effect varies in different conditions. For example, in bacteria *S. aureus*, the inhibition of H_2_S-synthesizing enzymes can potentially increase their vulnerability to immune defense and antibiotics [46, 47], indicating the protective role of the compound, whereas in a non-H_2_S-synthesizing bacteria *A. baumannii*, exogenous H_2_S improves the sensitivity of the bacteria to numerous antibiotics by targeting redox status and energy metabolism [48]. Regardless, these data imply that H_2_S plays a crucial role in bacteria survival, and exogenous H_2_S might promote the oxidative stress features in the host cell meanwhile inducing a similar or a protective effect in bacteria depending on the redox status (Figure 2).

### 3.2. Apoptosis

Apoptosis is a programmed mechanism involved in the regulation of body homeostasis by systematically killing cells that are no longer needed. The dysregulation of this process can lead to excessive cell death (e.g., in tissue fibrosis) or the vice versa (e.g., in cancer). Apoptosis is triggered through the activation of a group of protein known as caspases (Casp) in intrinsic- or extrinsic-dependent signaling pathways. Previous studies show that the treatment of H_2_S derived from either exogenous sources or a pathogenic oral bacteria *T. denticola* can significantly induce apoptosis in oral cells including the human periodontal ligament cells (PDLCs) and human gingival fibroblasts (HGFs) [49, 50]. The induction of apoptosis by oral H_2_S is mediated via mitochondria dependent pathway as evidenced by the promotion of Casp-3, Casp-8, Casp-9, cytochrome c, mitochondria depolarization, and the subsequent activation of p53 signaling cascade [51–55]. Moreover, the event is associated with the elevation of proapoptotic genes such as B cell lymphoma 2 (Bcl-2), phosphatase and tensin homolog, sirtuin, histone deacetylase, growth arrest, and DNA damage-inducible gamma, together with the ROS levels and DNA damage [56, 57]. However, the expression of the key component of death receptor apoptotic pathway, Casp-8, could not be affected by the increase, which suggests that the pathway is not necessarily targeted. In a recent study, the gingiva-derived mesenchymal stem cells (GMSCs) known to participate in immunomodulation and tissue regeneration have been shown to utilize CBS/CSE/H_2_S axis in mediating the apoptosis of regulatory T cell via the Fas/FasL signaling pathway [58]. With this crucial finding, it is essential to analyze the role of bacteria-derived H_2_S in the function of GMSCs both in health and disease states. Moreover, the abundance of several key H_2_S-producing bacteria in oral cavity noticeably relates with the apoptotic activities in the surrounding cells. A recent study analyzing the role of oral microbes in oral epithelial cells death found a positive correlation between the abundance of *S. gordonii*, *S. sanguinis*, and *P. gingivalis* with elevation of apoptosis and pyroptotic activities in a mechanism involving the elevation of Casp, TNF receptor p55, apoptosis-inducing factor (AIF), proteolytic activities of gingipain enzyme, cleaved poly (ADP-ribose) polymerase (PARP), and topoisomerase I, heat-labile protein-induced activation of interleukin-1*β*- (IL-1*β*-) converting enzyme and nuclear factor-kappa B (NF-*κ*B), and partial activation of protein kinase B (AKT)/mitogen-activated protein kinase (MAPK) cascades [59–64].

### 3.3. Inflammation

Inflammation is a response mechanism to tissue/cell damage and infection. In dental pulp mesenchymal stem cells and GMSCs, inflammation is associated with higher proliferation rate [65]. Besides, human gingival tissues from periodontal patients show improved expressions of inflammatory markers such as tumor necrosis factor-*α* (TNF-*α*), interferon gamma (IFN-*γ*), and interleukins (ILs) [66]. Inflammation strongly correlates with the decline in vitamin D; hence, an increase in vitamin D can suppress both pathogenic invasions and inflammatory responses in human gingival epithelial cells [67]. LPS from bacteria also induces the release of proinflammatories IL-6 and IL-8 in HGFs which maintain the release upon further treatment with LPS indicating lack of tolerance [68]. Mechanistically, it is suggested that *P. gingivalis* LPS binds to the Toll-like receptor 4 (TRL4) to mediate the downstream regulation of inflammatory activities [69]. In mouse abscess model, H_2_S from *P. gingivalis* has been reported to only enhance the inflammatory effect induced by CH_3_SH [70]. With respect to lifestyle, electronic cigarettes with flavorings are associated with high proinflammatory activities and oxidative/carbonyl stress in oral cells [71] and the use of fixed orthodontic devices with poor oral hygiene, high levels of H_2_S, and proinflammatory activities in children [72]. A previous study suggests that treatment with NaHS aggravates the proinflammatory activities of *P. gingivalis* in HGFs and PDLCs by activating the NF-*κ*B pathway [73]. Meanwhile, the treatment with GYY4137 in oral mucosa wound reduces the induced macrophage activation and restores the diminished H_2_S levels and prevents the polarization of macrophage 1, suggesting a potential anti-inflammatory influence of the slow-releasing donor [74]. Similarly, in HGFs, the treatment with diallyl sulfide significantly reduces the LPS-induced elevation of TNF-*α*, IL-1*β*, IL-6, and NF-*κ*B levels [75]. Overall, these data suggest that H_2_S may have pro or anti-inflammatory responses in oral cells. Although, the leading factors need to be further determined.

## 4. H_2_S and Oral Diseases

### 4.1. Oral Malodour (Halitosis)

**Figure 3:**
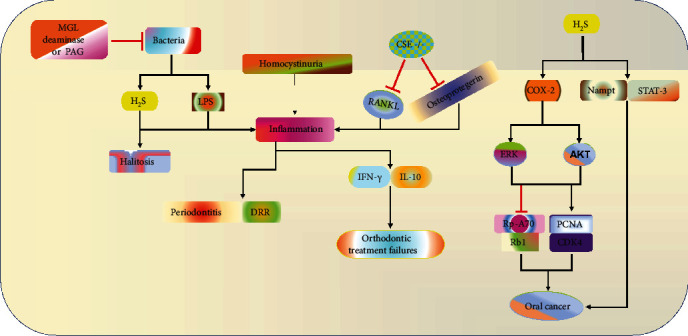
The summary of the mechanism and cellular markers targeted by H_2_S in regulating oral diseases. From left to right: H_2_S and LPS produced by oral bacteria participate in the promotion of halitosis and inflammation respectively. Homocystinuria also leads to elevated inflammatory responses thereby positively influencing oral diseases such as Periodontitis, DRR, and orthodontic treatment failures. However, inhibition of CSE enzyme significantly suppresses H_2_S and LP production, as well as inflammation by inhibiting RANKL and osteoprotegerin proteins. H_2_S also stimulates COX-2, Nampt, STAT-3, AKT/ERT pathways to facilitate cancer growth. MGL: L-methionine gamma lyase; PAG: D,L-propargyl glycine; H_2_S: hydrogen sulfide; LPS: lipopolysaccharides; DRR: dental root resorption; RANKL: receptor activator of nuclear factor kappa B ligand; COX-2: cyclooxygenase 2; Nampt: nicotinamide phosphoribosyltransferase; STAT-3: signal transducer and activator of transcription-3; IFN-*γ*: interferon gamma; IL-10: interleukin 10; AKT: protein kinase B; ERK: extracellular signal-regulated kinases; Rp-A70: replication protein A 70; Rb1: retinoblastoma protein 1; PCNA: proliferating cell nuclear antigen; CDK4: cyclin-dependent kinase 4.

Halitosis is a common medical condition of the oral cavity associated with the psychological and physical discomfort as a result of an offensive bad breath. H_2_S, (CH_3_)_2_S, and CH_3_SH are the main compounds causing the condition. Halitosis can be classified as intraoral or extraoral depending on the origin of the compounds. H_2_S and CH_3_SH are the common components of the former type, whereas (CH_3_)_2_S features the latter [76]. Extraoral halitosis can be further subdivided into blood-borne or nonblood borne originating from the respiratory tracts or blood, respectively. Intraoral halitosis is caused by several factors including oral bacteria and diseases [77]. Mimicking intraoral halitosis by treating rat epithelial cells with low concentrations of H_2_S gas for 50 days results in significant changes in cellular structure, vacuolization, and loss of intercellular matrix resembling halitosis in human [78]. An increase in the abundance of H_2_S-producing oral bacteria in oral biofilm has been associated with the disease [79]. In a recent study, both oral malodorous compounds (H_2_S, CH_3_SH, and (CH_3_)_2_S) and bacteria diversity have been reported to be higher in halitosis patients compared to normal individuals [80, 81]. Among others, the genera *Peptostreptococcus* and *Alloprevotella* together with the specie *Eubacterium nodatum* are highly abundant in halitosis patients and positively correlate with H_2_S and CH_3_SH concentrations in adults [82]. Similarly, in children with halitosis, evidences indicate that the rate of production/consumption of H_2_S is high as compared to healthy subjects [83]. However, the use of mouth-rinsing products could effectively reduce H_2_S levels in halitosis patients [84] (Figure 3). Together, these data show that oral bacteria are associated with halitosis through their involvement in the production of H_2_S. Also, H_2_S contributes immensely to the bad smell in halitosis and targeting this compound directly or indirectly might improve oral health and reduce the destruction of the oral tissues.

### 4.2. Periodontitis

**Figure 4:**
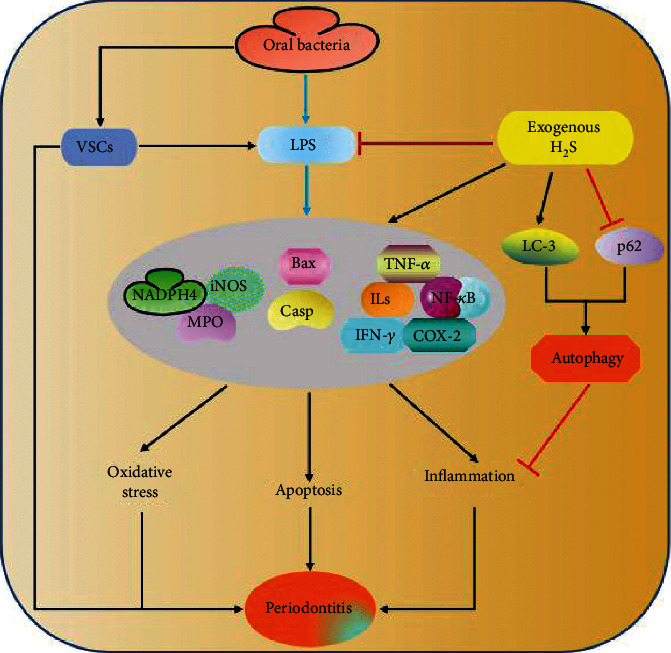
The illustration depicting the role of oral bacteria in periodontitis. VSCs and LPS produced by oral bacteria induce the promotion of inflammation, apoptosis, and oxidative stress by targeting several associated markers including ILs, COX-2, TNF-*α*, IFN-*γ*, NF-*κ*B, Bax, Casp, NADPH4, iNOS, and MPO. However, exogenous H_2_S suppresses LPS induced changes and triggers autophagy via LC-3 and p62 ultimately attenuating inflammation. H_2_S: hydrogen sulfide; LPS: lipopolysaccharides, VSCs; volatile sulfur compounds, NADPH4: nicotinamide adenine dinucleotide phosphate oxidase 4; MPO: myeloperoxidase; iNOS; inducible nitric oxide synthase; Bax: BCl-2-associated X protein; Casp: caspases, NF-*κ*B: nuclear factor-kappa B; ILs: interleukins, COX-2: cyclooxygenase 2; TNF-*α*: tumour necrosis factor alpha; IFN-*γ*: interferon gamma; LC-3: microtubule-associated protein 1A/1B-light chain 3.

Periodontitis is the common oral disease characterized by the chronic inflammation of the periodontal ligaments leading to the loss of connective tissue, alveolar bone resorption, and development of periodontal pockets. Oral bacteria play a major role in the development of this disease [85]. A qPCR analysis shows high abundance of *P. gingivalis*, *T. denticola*, and *T. socranskii* in plaque samples from aggressive (84, 74, and 71%) and chronic periodontitis patients (95, 94, and 89%) [86]. Antibacterial treatments inhibiting the growth of *P. gingivalis* have been shown to be effective in combating the disease in clinical trials [87, 88]. Similarly, periodontitis is also correlated with oral malodour in patients' model [89]. In periodontitis patients, H_2_S levels show positive association with the abundance of *P. gingivalis* in tongue coatings [90], and the bacteria growth together with volatile smell in the oral cavity can be suppressed with methionine gamma-lyase deaminase/CSE inhibitor PAG [91]. CBS deficiency specifically causes a condition known as homocystinuria, which is characterized by elevation of proinflammatories such as IL-1b, IL-6, IL-8, and TNF-*α*. A recent study aiming to compare the periodontium of the CBS^+/-^mouse model to the wild type suggests a significant correlation between periodontal diseases and CBS deficiency [92]. Moreover, another study reports that supplementation of H_2_S using GYY4137 promotes inflammatory and autophagic responses in LPS-treated HPDLCs and ligature-induced rats [93]. Here, GYY4137 treatment could markedly elevate the expressions of Bcl-1 and LC-3 and decrease that of p62, whereas the inhibition of the autophagy with 3-methyladenine further aggravates inflammatory activities, implying that the treatment triggers a protective autophagy in order to avert the enhanced inflammation. However, another study suggests that a H_2_S-releasing ketoprofen drug, ATB-352, can prevent the LPS-induced periodontitis and associated bone resorption in rats by reducing inflammation, apoptosis, and ROS through attenuating the IL-1*β*, TNF-*α*, NF-*κ*B, Bax, cyclooxygenase-2 (COX-2), and iNOS expressions, myeloperoxidase activities, and tartrate-resistant acid phosphatase positive cells as well as upregulating Bcl-2 [94]. This is consistent with the previous studies conducted using H_2_S donors, ATB-346, and Na_2_S in periodontic rat model which demonstrates the reduction of proinflammatory activities, ROS, and bone loss [95]. Meanwhile, NaHS treatment could not show any reduction or promotion of bone loss in ligature-induced rats [96, 97], although the presence of both nitric oxide (NO) and H_2_S moiety in ketoprofen derivatives might be the reason for the observed anti-inflammatory property. The available information suggests that the nature of the donor influences their effects, and leaves the question of the role of H_2_S on periodontitis unanswered. But it is possible that H_2_S produced by bacteria can facilitate periodontitis; however, more studies are needed to examine the mechanisms involved (Figure 4).

### 4.3. Dental Root Resorption (DRR)

DRR is the medical condition featured by the mechanical- or chemical-induced loss of the protective tissues of the root apex structure of the tooth which exposes the tissues to bacterial infections [98, 99]. One of the common causes of DRR is orthodontic treatments, although in most cases, the condition is classified as minor or moderate. Without further stimulation or persistent inflammation, the RR can be routinely repaired [100]. Oral bacteria and their byproducts such as H_2_S promote inflammation and in that sense enhance the progression of DRR. To examine the influence of H_2_S in DRR, Lu et al. used a CSE-knockout mouse model and compared them with the wild type. The results indicate that the downregulation of CSE, which is the main H_2_S-producing enzyme in osteoclast, attenuates the progression of orthodontic RR [101]. Simultaneously, the reduction in mRNA levels of the RANKL and osteoprotegerin which have previously been associated with proinflammatory responses in orthodontic RR could be observed in CSE knockout mice [101, 102]. This confirms that the increase in H_2_S may promote the progression of the condition. However, further studies are needed to examine the effect of exogenously produced H_2_S in the disease.

### 4.4. Gingivitis

Gingivitis is an inflammatory disease primarily caused by the deposition of microbial plaque near the gingival sulcus [103]. The disease is associated with the abundance of *Streptococcus*, *Fusobacterium*, *Actinomyces*, *Treponema*, *Capnocytophaga*, and *Bacteroides.* On the other hand, the healthy gingival is characterized by species such as *Streptococcus sanguis* and *Fusobacterium naviforme.* Gingivitis occurs in two forms: acute necrotizing ulcerative and chronic gingivitis; however, chronic form is the most common one. In an earlier study, the accumulation of dental plaque has been determined to be much greater in older individuals that younger ones possibly due to poor oral hygiene [104]. It has been reported that oral VSCs in dogs with gingivitis have a significant relationship with the amount of plaque and the severity of the disease [105]. In addition, gingival inflammation and bleeding on probing also correlate with sulfide levels in human gingival mucosae [106, 107]. Therefore, the elevation of sulfide levels as a result of the accumulation of pathogenic bacteria in gingivitis can positively influence the disease progression by promoting gingival inflammation.

### 4.5. Oral Cancer

#### 4.5.1. Oral Squamous Cell Carcinoma (OSCC)

OSCC is most frequently diagnosed type of head and neck carcinoma with recent global estimation of 377,713 new cases and 177,757 deaths in 2020 [108]. Despite a recent increase in incidence rate [109], the disease has a relatively stable survival rate which increased for about 8.4% from 1980s to 2010s [110]. Some of the common risk factors for the disease include excessive smoking, alcohol abuse, and oral diseases. Otherwise, oral bacteria have also been identified to be an independent risk factor for the disease in nonsmokers and oral human papillomavirus- (HPV-) negative patients [111]. Generally, in OSCC patients, key bacteria including *Prevotella*, *Fusobacteria*, *Pseudomonas aeruginosa*, *Haemophilus influenza*, *Campylobacter*, *Parvimonas micra*, and *Filifactor alocis* are distinctly elevated, correlate with the stages of OSCC progression, and act on vital signaling cascades [112–116].

The analysis of punch biopsies and benign mucosae reveals that H_2_S is significantly upregulated in OSCC patients as compared to the control group as evinced by the increase of CSE, CBS, and 3-MST levels [117]. In addition, OSCC also contains higher levels of procarcinogenic markers such as phosphorylated signal transducer and activator of transcription-3 (p-STAT3), mitoNEET, telomerase reverse transcriptase, and MAPK. Besides, the extreme volatile malodor has also been reported in head and neck carcinoma patients and suggested to be a potential diagnostic target for the diseases, which further confirm a decisive relationship between these volatile compounds and the disease [118, 119]. It has been shown that surgical treatment of OSCC can effectively reduce the volatile malodor including those of sulfide containing compounds commonly generated by oral bacteria [120, 121]. Using a donor NaHS, previous studies suggest that the exogenous H_2_S promotes the proliferation and cell cycle progression in OSCC cell lines Cal27, GNM, and WSU-HN6 through elevating the expressions of proliferating cell nuclear antigen and cyclin-dependent kinase 4 and reducing those of replication protein A 70 and retinoblastoma protein 1 via the AKT/extracellular signal-regulated kinase 1/2 (ERK1/2) pathways [121, 122]. Together, these data confirm the involvement of oral bacteria and their products including H_2_S in the progression of OSCC and illuminate the potential of inhibiting the production of H_2_S in combating this disease.

#### 4.5.2. Oral Adenoid Cystic Carcinoma (OACC)

OACC is the rare form of head and neck carcinoma of unknown etiology. The statistics show a decline in the prevalence of the disease from 1970s to 2000s [123]. Despite having a relatively high short-term overall survival which ranges from 90% in 5 years to 69% in 15 years, the disease has high recurrence rate [124, 125]. Although not 100% effective, both surgery and radiotherapy can significantly impede the progression of the disease [126]. The analysis of oral bacteria composition between OACC patients and healthy individuals indicates a considerable difference in genera *Streptococci*, *Neisseria*, and *Porphyromonas* [127]. In a case study of a single, 54-year-old female OACC patient, the protein expressions of the three H_2_S-synthesizing enzymes as well as those mitoNEET and nicotinamide phosphoribosyl transferase have been reported to be upregulated in OACC tissues as opposed to adjacent benign oral mucosae; however, the decrease in the production of H_2_S for over 30% could also be observed in the OACC samples, indicating that the H_2_S is overutilized in the disease model and might be involved in the progression of the disease [128]. So far, little is known on the role of oral bacteria and oral malodour in the development of OACC. Even though the available information suggests the involvement of H_2_S in the progression of the disease, the influence of exogenous H_2_S demands further exploration.

#### 4.5.3. Oral Cavity Mucoepidermoid Carcinoma (MEC)

MEC is one of the least-researched cancers but a highly prevalent salivary gland malignancy. The disease has relatively favourable prognosis; however, advanced age, advanced stage, and high-grade tumors negatively impact the survival rate [129, 130]. Surgery is the common treatment option for the disease. In a single-case study involving a 55-year-old woman, the expressions of CSE, CBS, and 3-MST have been reported to be elevated in MEC tissues; meanwhile, the levels of free H_2_S, acid labile, and bound sulfane sulfur remain the same between MEC and neighboring benign oral mucosae [130]. In addition, the study reported the elevation of key markers such as phospho-ser727-STAT-3 and Nampt that are known to promote cancer growth and metastasis as well as interact with H_2_S-synthesizing enzymes [131, 132]. Furthermore, the antiapoptotic and antiautophagic protein mitoNEET has also reported to be upregulated in the metastatic tissue as compared to benign [133]. Collectively, this information indicates that H_2_S is highly produced and utilized in MEC and plays a crucial part in the progression of the disease. However, limited information is available on the matter, and more studies are needed to deepen the exploration.

### 4.6. Endodontic Treatment Failures

Endodontic treatment incorporates surgical and nonsurgical treatment options for root canal [134, 135]. The therapy involves the treatment of the infection, removal of the invading microorganisms, and perfect sealing of the canal. Despite the success of the method used, in significant cases, the treatments have been reported to fail. Some of the factors causing the failure of the therapy as identified in patients from Japan include perforation, root fracture, open apices, periodontic diseases, fenestrations, and accessory canal [136]. Apart from these factors, another key causative of endodontic failures is bacterial infection [137]. A substantial difference has been reported in patients with failed treatment as opposed to the untreated ones, with the former featured by the dominance of *Enterococcus faecalis* [138]. Also, bacteria such as *P. gingivalis* and *F. nucleatum* have been reported to participate in the treatment failure. With respect to H_2_S, previous studies indicate that VSCs specifically H_2_S and CH_3_SH can trigger proinflammatory responses in endodontic treatment failures by increasing the levels of IFN-*γ* and IL-10 in patients [139, 140]. This suggests that H_2_S produced by oral bacteria can potentially increase inflammation which in turn hinders the treatment efficacy.

## 5. Conclusion

H_2_S is among the VSCs released by the oral microbes and strongly produced by oral cells. The upregulation of H_2_S production as result of endogenous/cellular mechanisms or exogenous/bacteria activities has significant impact in oral health. This is due to the role of H_2_S in regulating cellular activities such as oxidative stress, apoptosis, cell differentiation, and inflammation. In most oral diseases, H_2_S is a prerequisite for further progression and severe conditions. Besides, the reducing power of H_2_S helps to suppress the effects of drugs that work primarily through promotion of oxidative stress; this is a crucial mechanism observed in antibiotic resistance by the oral bacteria. In recent years, the role of H_2_S has been well documented in various diseases including cancer, heart diseases, respiratory diseases, and metabolic diseases [141–144]. Despite high production of this gas by pathogenic oral bacteria, yet few information is available concerning the matter. In chronic oral diseases such as cancers, high production and high utilization of H_2_S have been reported to the extent that cancer tissues and surrounding tissues have no significant difference in H_2_S levels despite high levels of the synthetase enzymes observed in cancer tissues. Also, few clinical trials are available on the subject and none of them specifically targeted H_2_S alone which stresses the need for further studies to be conducted (Table 1). Therefore, it is important to examine the role of H_2_S in oral diseases in order to establish literature foundation for the possibility of using this gasotransmitter as diagnostic tool or therapeutic target.

Additionally, treatment of oral diseases with H_2_S donors has also been shown to have conflicting outcomes; this effect is possibly in relation to the nature of the donor used and their mechanism of actions. With regard to this, it is crucial to determine the impact of downregulation of H_2_S levels in these disease models and check the possibility of combining H_2_S inhibitors and other treatment options for oral diseases in order to improve the sensitivity of the therapies. One of the challenges facing the inhibition of H_2_S in oral diseases especially the H_2_S produced by oral bacteria is the complexity of their mechanisms. Different bacteria can produce the gas through different enzymes which affects the specificity of the available inhibitors. With further research, many challenges facing this venture will be solved. Hence, it is indispensable to examine the mechanism used by H_2_S to induce its effect in oral diseases, cellular activities targeted, and outcome. Otherwise, the future advance in this field will help to clarify and improve the current knowledge available concerning H_2_S and oral diseases.

## Figures and Tables

**Table 1:** Some of the clinical trials targeting VSCs in treating oral diseases.

Treatment option	Disease	Effects	References
Chlorhexidine	Halitosis	Reduces H_2_S-producing bacteria as well as H_2_S levels	[145–150]
Colgate 360			
Triclosan/copolymer/dentifrice			
Rinsing or drinking of water			
Hinokitiol-containing gel			
Pycnogenol			
YAG laser irradiation	Periodontitis	Reduces VSCs	[151]
Antiplaque dentifrices	Gingivitis	Reduces VSCs and proinflammatories	[152, 153]
Oral prophylaxis such as tongue scraping			

## Abbreviations

VSCs: Volatile sulfur compounds
LPS: Lipopolysaccharides
H_2_S: Hydrogen sulfide
CSE: Cystathionine gamma-lyase
CBS: Cystathionine beta-synthase
3-MST: 3-Mercaptopyruvate sulfur transferase
PLP: Pyridoxal 5′-phosphate
GCF: Gingival crevicular fluid
CH_3_SH: Methyl mercaptan
(CH_3_)_2_S: Dimethyl sulfide
L-Cys: L-Cystine
L-Hcy: L-Homocysteine
Cd: Cysteine desulfurase
OASS-A/-B: O-Acetylserine sulfhydrylase-A/-B
CBL: Cystathionine beta-lyase
LS: Lanthionine synthase
MGL: L-Methionine gamma-lyase
DNA: Deoxyribose nucleic acid
H_2_O_2_: Hydrogen peroxide
ROS: Reactive oxygen species
GSH: Glutathione
NaHS: Sodium hydrosulfide
Casp: Caspase
PDLCs: Periodontal ligament cells
HGFs: Human gingival fibroblasts
Bcl-2: B cell lymphoma 2
GMSCs: Gingiva-derived mesenchymal stem cells
LC-3: Microtubule-associated protein 1A/1B-light chain-3
AIF: Apoptosis-inducing factor
PARP: Poly (ADP-ribose) polymerase
TNF: Tumor necrosis factor
TLR4: Toll-like receptor 4
NF-*κ*B: Nuclear factor-kappa B
ILs: Interleukins
AKT: Protein kinase B
MAPK: Mitogen-activated protein kinase
NO: Nitric oxide
DRR: Dental root resorption
OSCC: Oral squamous cell carcinoma
HPV: Human papillomavirus
STAT3: Signal transducer and activator of transcription-3
IFN-*γ*: Interferon gamma
COX-2: Cooxygenase-2
OACC: Oral adenoid cystic carcinoma
ERK1/2: Extracellular signal-regulated kinase 1/2
MEC: Mucoepidermoid carcinoma
